# Irritability in the Mix: An Exploratory Study of the Role of Irritability in Characterising Mood Disorders

**DOI:** 10.1111/bdi.70185

**Published:** 2026-09-30

**Authors:** Erica Bell, Maree Inder, Gin S. Malhi, Richard Bryant, Philip Boyce, Richard J. Porter

**Affiliations:** ^1^ Academic Department of Psychiatry, Kolling Institute, Northern Clinical School, Faculty of Medicine and Health The University of Sydney Sydney New South Wales Australia; ^2^ CADE Clinic, Royal North Shore Hospital, Northern Sydney Local Health District Sydney New South Wales Australia; ^3^ Department of Psychological Medicine University of Otago Christchurch New Zealand; ^4^ Department of Psychiatry University of Oxford Oxford UK; ^5^ Oxford Uehiro Centre for Practical Ethics, Faculty of Philosophy University of Oxford Oxford UK; ^6^ School of Psychology University of New South Wales Sydney New South Wales Australia; ^7^ Speciality of Psychiatry, Sydney Medical School, Faculty of Medicine and Health University of Sydney Sydney New South Wales Australia

**Keywords:** bipolar disorder, irritability, mixed states, phenomenology, psychological treatment

## Abstract

**Objectives:**

To examine the relationship between irritability and mixed states in bipolar disorder to determine how subjective irritability and mixed symptoms of mania and depression manifest in patients with bipolar disorder over a period of 18 months.

**Methods:**

This exploratory analysis examined longitudinal data from a randomised controlled trial of a psychological intervention for patients with bipolar disorder. Scores of subjective irritability and mood morbidity were analysed from data sampled over ten timepoints over the trial from 82 patients.

**Results:**

Significant associations were found between the presence of irritability and mixed symptoms (*F*(1, 684) = 20.468, *p* < 0.001). This relationship remained significant throughout the duration of the study and the administration of a psychological treatment which reduced both ratings of mood and irritability. Notably, when mixed symptoms are absent, irritability nevertheless remained for nearly half the time.

**Conclusions:**

Irritability and mixed symptoms are phenomenologically linked. The persistence of this link, regardless of the existence of mixed symptoms, suggests that irritability permeates bipolar disorder and is core to its phenomenology. Future studies should interrogate the tethering of mixed symptoms and irritability in the context of bipolar disorder as this may lead to a deeper understanding of the core characteristics of the illness—both clinically and in terms of its neurobiology.

## 1 Introduction

### 1.1 Irritability as a Psychiatric Phenomenon

Within psychiatry, irritability is a transdiagnostic phenomenon and a common clinical complaint among patients [1, 2, 3]. It is mentioned in over 30 different disorders within the Diagnostic and Statistical Manual of Mental Disorders—5th Edition Text Revision (DSM‐5‐TR) [4], serving as a feature and/or a diagnostic criterion. This ubiquity has led some to advocate for irritability disorders in adulthood to be considered a category of their own [5]. Indeed, irritability is so commonplace that its relationship to mental illnesses has been described as analogous to the relationship between fever and physical illnesses in general medicine [6]. This is because it is non‐specific, and its manifestation does not necessarily point to an underlying cause or mechanism. At the same time, its presence is often a strong signifier of illness and in practice it suggests that a person may require medical attention—hence it is an important indicator of illness.

Although definitions of irritability vary, and there is no consensus [7], a recent working definition is that irritability is a mood characterised by a heightened sensitivity to stimuli coupled with a lowered threshold for responding with anger or aggression to typically less vexing stimuli [7, 8]. It is often seen in the context of unmet needs (e.g., hunger, sleep), illness or somatic complaints (e.g., hormonal fluctuations) [9, 10] and in phases of life in which the capacity to regulate one's emotions and moods is compromised (e.g., young childhood, dementia and Huntington's disease) [11, 12, 13]. Nevertheless, its nature within the context of psychiatric disorders remains largely unmapped. One hypothesis is that, rather than being a symptom of such a broad array of conditions with purportedly distinct aetiologies, it is an epiphenomenon that signals when there is a problem per se, but remains non‐specific to any underlying cause. In other words, irritability manifests when there is an underlying problem, but its manifestation is not specific to the underlying mechanisms that drive the problem, and irritability may instead be a secondary by‐product of this process. This possibility is worthy of further exploration, and just as fever is a useful clinical indicator, irritability too may serve as a broad signal for an underlying psychiatric condition. In this way, it may alert both patients and clinicians to an insidious problem before it crystallises and manifests acute symptoms.

### 1.2 Irritability in Mood Disorders

Within our current major diagnostic taxonomies, the DSM‐5‐TR and the International Classification of Diseases 11th Revision (ICD‐11) symptoms of depression and mania have been separated and conceptualised as groups of symptoms from two diametrically opposed poles of mood [4, 14]. However, irritability defies this separation in that it is listed within both sets of symptoms—as a symptom of mania in adults, and as a symptom of depression in children and adolescents. In addition, within DSM manic episode diagnostic criteria, if the prevailing mood during a manic episode is irritable (as opposed to euphoric), then there is a higher threshold required before a patient can be diagnosed with mania (four symptoms as opposed to the usual three) [4]. This distinct requirement and separation of irritability from other symptoms of mania perhaps indicates a tacit acknowledgment of irritability's lack of specificity. And whilst it is common enough in the context of bipolar disorder to be listed as a ‘symptom’ of mania, it is *not* specific enough to be counted as a mood that is ‘equal’ to euphoria.

The higher threshold required of irritability is puzzling, as irritability is considered so central to manic symptomatology that it was listed as a key clinical feature (along with elevated mood and grandiosity, hyperactivity, pressured speech) in psychiatric textbooks published in the first half of the 20th century [15]. The prevalence of irritability among clinical presentations of mania has meant that its co‐occurrence with depressive symptoms is usually classified as a mixed presentation or episode. Indeed, in mixed depression, that is depressive episodes with so‐called ‘contra‐polar’ symptoms, irritability is considered one of the most prevalent (manifesting in approximately 30%–70% of presentations [16, 17]) and is therefore classified as a key symptom of mixed states [16, 17, 18]. Thus, whilst irritability is in many ways non‐specific, it is at the same time characteristic of bipolar disorder presentations [19]. Therefore, the exact nature of irritability and its relationship to bipolar disorder as we currently understand it presents a quandary worthy of further exploration and characterisation.

### 1.3 The Status of ‘Mixity’ in Bipolar Disorder

Whilst current diagnostic taxonomies remain largely wedded to the separation of manic and depressive symptoms, it is well‐established that clinically, many patients experience mixed symptoms (co‐occurring depressive and manic symptoms; also known as mixity [20]), with recent estimates ranging from 12%–28% of patients with a mood disorder experiencing mixed features [21]. The ability to classify mixed presentations as an ‘episode’ in and of themselves is a key point of distinction between DSM‐5‐TR and ICD‐11. In the latter, an episode of illness can be classified as a mixed episode if there are several ‘prominent’ symptoms from both depression and mania co‐occurring or alternating rapidly [14]. In DSM however, since the 5th edition in 2013, mood episodes can only be coded as depressed or manic, with contrapolar symptoms being coded as mixed ‘features’, thus minimising their importance [4, 22]. Further, in DSM‐5, irritability cannot be counted as a manic/hypomanic symptom towards a mixed features specifier for a depressive episode. This inexplicable discrepancy between the centrality of irritability to mania and its absence within the mixed features criteria points towards a lack of clarity firstly, as regards the clinical characteristics of mixed states and secondly, the relationship between irritability and bipolar symptomatology.

Therefore, our entire conceptualisation of bipolar disorder as being defined by mania, with or without depression, is challenged by the existence of mixed states [23]. The prevalence and the general lack of clinical and academic recognition of mixity in bipolar disorder has meant that mixed states are poorly understood [24]. This is reflected in outcomes for patients, with mixity being associated with poorer prognosis and response to treatments and an increased risk of impaired functionality and suicide [18, 25]. Thus, advances in our understanding of mixity in bipolar disorder are urgently required to improve outcomes for patients through better detection, diagnosis and treatment.

### 1.4 Characterising Irritability and Bipolar Disorder

In addition to mixed states comprising a significant proportion of bipolar presentations, in clinical practice, patients with bipolar disorder spend the majority of their time either in subsyndromal, euthymic phases of illness, or in periods between them, especially prior to the onset of the illness [26, 27]. Thus, clear‐cut ‘pure’ forms of mania and depression form the minority of experiences for patients with bipolar disorder. Indeed, even within these pure forms, there are underlying factors of mixity; for example, 12%–29% of patients with pure mania show depressive symptoms [28, 29]. Therefore, if we wish to improve our understanding of the illness it is imperative to identify the clinical symptoms that characterise the illness and can also be used for early detection, treatment and prevention. To this end, irritability provides a promising lead; it spans from normality through to the acute phases of illness and, at the same time, appears to be a key feature of mixity. Taken together, irritability may provide a potential avenue for early detection and management (see Figure 1).

**FIGURE 1:**
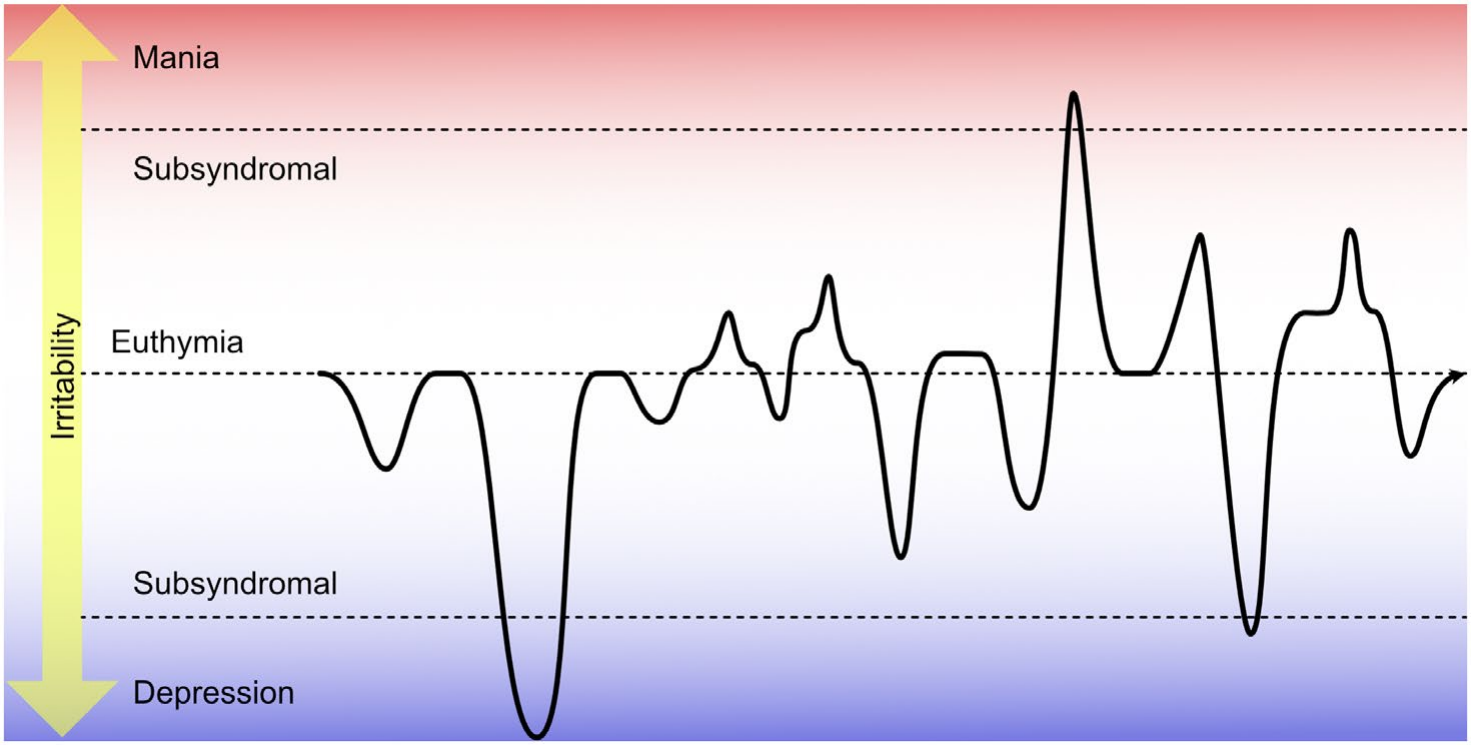
Irritability across the mood spectrum and example life chart of bipolar illness. This schematic illustrates how irritability is present across the mood spectrum, from the acute symptoms of mania (red) and depression (blue), as well as during subsyndromal and euthymic periods. These phases are demarcated using dashed lines. An example life chart of illness is shown by a solid line. This life chart illustrates how for most patients with a mood disorder, the time spent during acute phases of illness is small compared to the periods spend with subsyndromal, mixed symptoms and euthymia. Whilst the acute periods of illness may be the focus of management in terms of both symptomatic treatment and prophylaxis, mixed or subsyndromal symptoms are often overlooked.

Another advantage of pursuing irritability as opposed to many other symptoms of bipolar disorder is that aside from being a common feature of the illness, it retains its appearance throughout the mood spectrum—manifesting as a consistent feature in different phases of the illness, as well as being present in subsyndromal periods (see Figure 2). In other words, symptoms can change in their appearance over the mood spectrum, such as psychomotor activity being excessive in mania or slowed in depression. Other symptoms manifest in one pole of the illness and may be absent in the other; for example, libido is increased during mania but diminished or absent altogether in depression. Irritability, on the other hand, is increased in both acute exacerbations of mood (mania and depression), and therefore can be measured in the same way across the phases of illness.

**FIGURE 2:**
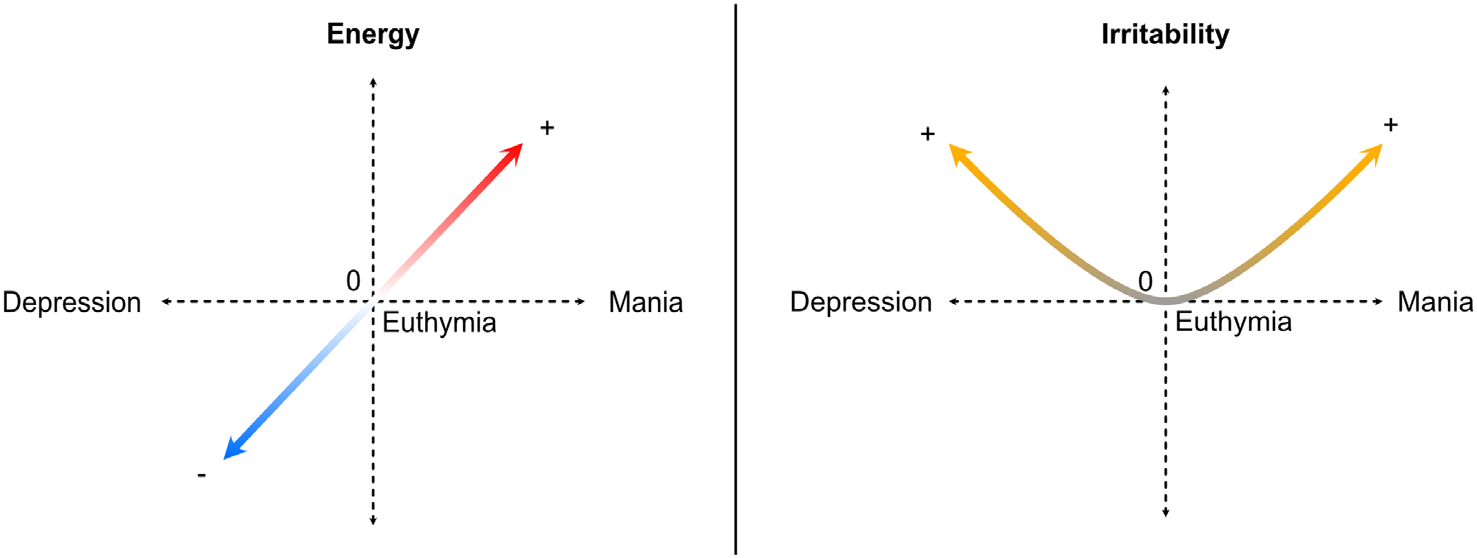
Schematic illustrating the relationship between other symptoms of mood disorders and irritability. The left panel uses the example of energy to illustrate how symptoms such as energy, appetite, sleep, motivation and drive, and cognitive symptoms all have a linear relationship with mood. That is to say, each symptom is deficient in depression and excessive in mania. However, irritability shows a non‐linear relationship with the poles of mood, wherein it is excessive in both depression and mania, and absent during euthymia. Thus, irritability retains its ‘shape’ across the mood spectrum and manifests in the same way whether it is appearing in the context of depression, mania or in between.

In clinical practice, mixed states, which have been largely overlooked to date—are present for much of the time and it is here that irritability is likely to be found [18]. Therefore, in this study we examined a dataset to investigate our hypothesis that irritability is a core feature of bipolar disorder—because it is central to mixed states and spans subsyndromal presentations, which are the more consistent clinical state of the illness.

### 1.5 Aims

In this preliminary analysis, we aimed to characterise the relationship between mixity and irritability in a population of patients with bipolar disorder. We hypothesise that irritability and mixity will be significantly correlated, suggesting a relationship between the manifestation of irritability in bipolar patients and the presence of mixed symptoms.

## 2 Methods

This study utilised measures of irritability obtained from data from a psychological therapy trial which recruited adolescents and adults with bipolar disorder [30]. Detailed methodology is provided in the publication of this randomised controlled trial (RCT) of Interpersonal and Social Rhythm Therapy (IPSRT) versus a ‘control’ psychological intervention of Specialist Supportive Care (SSC) over 18 months [30]. Ethical consent for the original study and any subsequent analysis was obtained from the Canterbury Ethics Committee, and the trial was registered with the Australian and New Zealand Clinical Trials Registry (ACTRN12605000722695). Here, we summarise the key aspects pertinent to the current analysis.

### 2.1 Measures

After obtaining consent, and prior to randomisation, the treating psychiatrist completed an Axis I diagnostic assessment using the Structured Clinical Interview for DSM‐IV (SCID) [31]. Throughout the 78‐week study, participants completed a series of questionnaires at 10 time points approximately every 9 weeks. Therefore, measurements were taken at weeks 0 (baseline), 8, 17, 26, 34, 43, 52, 61, 69 and 78.

The questionnaires included a modified version of the self‐report Symptom‐Checklist 90 (SCL90), a 90‐item comprehensive psychological assessment of a broad range of psychopathology [32]. This assessment asks ‘During the past week, how much were you bothered by…’ for each item listed, which can be scored from 0 (not at all) to 4 (Extremely). Within the hostility subscale [33] there is an item that measures subjective irritability (item 11 – ‘Feeling easily annoyed or irritated’). This item was utilised as the irritability measure for the purposes of the current analysis.

In order to chart the course of illness over the study period, participants completed the Longitudinal Interval Follow‐up Evaluation (LIFE) every 6 months. The LIFE was developed to retrospectively assess the longitudinal course of psychiatric disorders at regular intervals. The LIFE allows clinicians and researchers to assess the dates of individual episodes of a disorder and to rate the overall burden of symptoms. The validity of the LIFE in retrospectively determining mood state on a weekly basis has been demonstrated using the same cohort as the current analysis [34].

The LIFE asks participants to retrospectively rate their burden of mood symptoms on a weekly basis in an interview format, conducted at 6‐month intervals. Participants rated their mania and depression symptoms separately on a 0–5 scale where 0 = no symptoms, 1 = residual symptoms, 2 = partial remission, 4 = does not meet DSM criteria but has major symptoms or impairment and 5 = fulfils definite criteria for an ‘episode’ with the presence of either extreme impairment in functioning or psychotic symptoms. Ratings were carried out over the telephone by a trained research assistant who was blind to the treatment condition. For the current analysis, LIFE scores for depressive and manic symptoms for the 4 weeks immediately preceding and following each timepoint were calculated to correspond with the sampling of SCL‐90 scores. Figure 3 shows the timing of the collection for both the SCL and LIFE scores.

**FIGURE 3:**
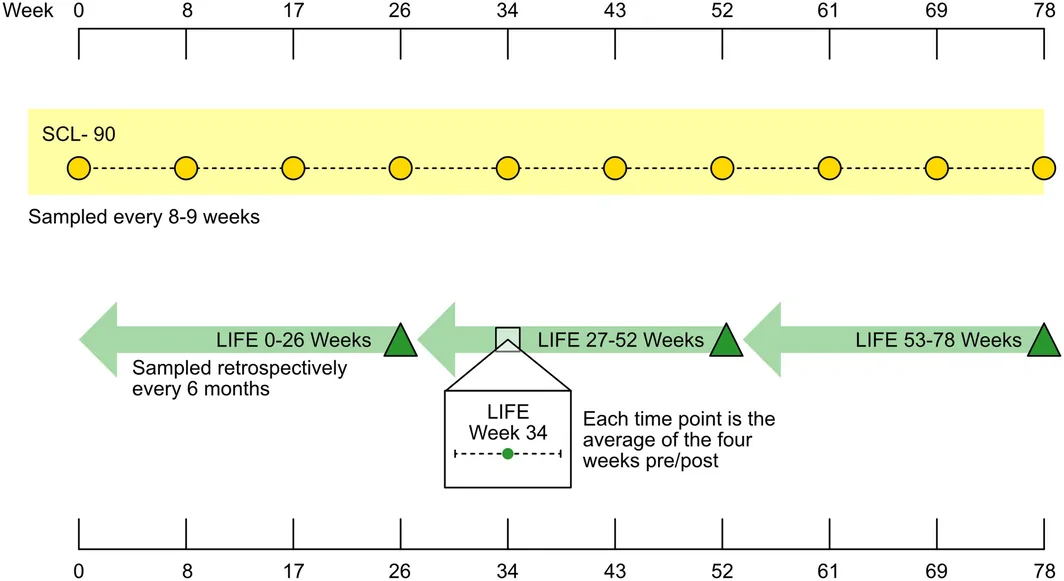
Timing of the Symptom‐Checklist 90 (SCL‐90) and Longitudinal Interval Follow‐up Evaluation (LIFE) over the study. The SCL‐90 was completed every 8–9 weeks by the participant. The LIFE was completed approximately every 6 months and retrospectively rated the burden of depressive and manic symptoms per week for the preceding 6 months. For the current analysis, average scores of depressive and manic symptom burden for each time point were calculated by averaging the scores for the 4 weeks preceding and following each timepoint.

A measure of ‘mixity’ was derived from the LIFE scores at each timepoint, wherein if a patient scored ≥ 0.5 for both depressive and manic symptoms at the same timepoint, mixity was determined to be present. A threshold of 0.5 was set as this would mean a patient would have had at least residual symptoms of both mania and depression for half of the sampling time for each timepoint (4 weeks pre and post). As such, the mixity variable for each timepoint was either coded as 1 = present or 0 = absent.

### 2.2 Participants

The study recruited 100 adolescents and young adults aged 15–36 years with Bipolar I, Bipolar II and Bipolar Disorder Not Otherwise Specified, with an adequate understanding of English, to participate in psychotherapy (see Table 1). Written informed consent was obtained from all participants, and they were recruited from a range of services including mental health services and General Practitioners. Exclusion criteria were schizophrenia or schizoaffective disorder and severe alcohol or drug dependence.

**TABLE 1:** Clinical characteristics of the study participants.

Age (M ± SD)	27.20 ± 5.82
Female (%)	*n* = 64 (78%)
Bipolar Diagnosis	Bipolar I—*n* = 66
	Bipolar II—*n* = 14
	Not Otherwise Specified—*n* = 2

Eight four (84%) of the participants completed the study. Across both treatment groups, the reasons for failure to complete were: moving from the study location (*n* = 6), failure to engage (*n* = 1), withdrawal (*n* = 3), requiring alternative treatment (*n* = 2), death by suicide (*n* = 1), and lost to contact (*n* = 3) [30]. Of this group, 82 had adequately complete data sets of both irritability and LIFE scores for inclusion in this analysis.

### 2.3 Statistical Analysis

All analyses were performed using IBM SPSS Statistics (v.29 for Mac). Previous analyses in this cohort determined there were no significant differences between treatment groups in measures of irritability and mood morbidity at baseline and Week 78 [30, 35]. As such, participants were not separated according to treatment type for the purposes of this analysis.

To interrogate whether there is a correlation between irritability and mixity status, a univariate ANOVA was performed with irritability (SCL90 item 11) as the dependent variable. Time and diagnosis were included as fixed factors and participant identification (ID) number was added as a random factor. In addition to examining each main effect, two interaction terms were included in the final model; these consisted of a diagnosis‐by‐ID interaction term (not reported due to lack of clinical relevance) and a mixity‐by‐time interaction term. All assumptions to perform an ANOVA were satisfied.

Secondly, to corroborate the results of the ANOVA, a Chi‐square test was utilised to examine the relationship between mixity status and the presence of irritability. To do this, an irritability threshold variable for each timepoint was set at ≥ 1, wherein if a patient scored ≥ 1 on SCL‐90 item 11 for any timepoint, irritability was coded as present. This is because a score of 1 corresponds to being bothered ‘a little bit’ by irritability over the past week including on the day of assessment. Therefore, irritability must have been present to some degree. If scores were < 1, irritability was coded as absent for that timepoint.

## 3 Results

Total scores on the SCL90 item 11, LIFE depression and mania scores significantly reduced from baseline and Week 78 (i.e., after long‐term IPSRT or SSC). These results are shown in Table 2, with significance calculated via a paired‐samples *t*‐test.

**TABLE 2:** Descriptive statistics of irritability, depression and mania symptom scores from baseline to Week 78.

	Baseline	*N*	Week 78	*N*	*t*
SCL90 item 11	1.75 (1.34)	81	0.96 (1.20)	78	3.91**
LIFE Depression mean	1.92 (1.14)	82	0.79 (0.99)	71	6.20**
LIFE Mania mean	0.73 (0.78)	82	0.31 (0.55)	71	3.94**

*Note:* Variation in *n* is a result of incomplete measures of SCL90 item 11, LIFE depression or mania scores for baseline or week 78.

** *p* < 0.001 (two‐tailed).

### 3.1 Mixity

Across the 82 participants, each with 10 timepoints of data, approximately 30% of all timepoints recorded mixity as being present. Of all participants, 65 (79%) had at least one timepoint with mixity present, and 22 participants (27%) had mixed symptoms for at least half of the 10 timepoints. Thus, there were a sufficient number of timepoints across the participants with mixity, allowing for examination of the relationship between mixity and irritability.

### 3.2 Relationship Between Irritability and Mixity

There was a significant main effect of mixity (*F*(1, 684) = 20.468, *p* < 0.001), and time (*F*(1, 684) = 4.435, *p* < 0.001) on scores of irritability. There was no significant main effect of diagnosis (*F*(2,79.427) = 0.650, *p* = 0.525) and no significant interaction between mixity and time (*F*(9,675) = 0.592, *p* = 0.791) on irritability scores.

When examined using a chi‐square test, the presence of mixity was associated with a significantly greater proportion of irritability, to no irritability, than when mixity was absent (*χ* ^2^ = 57.691, df = 1, *p* < 0.001) (see Figure 4).

**FIGURE 4:**
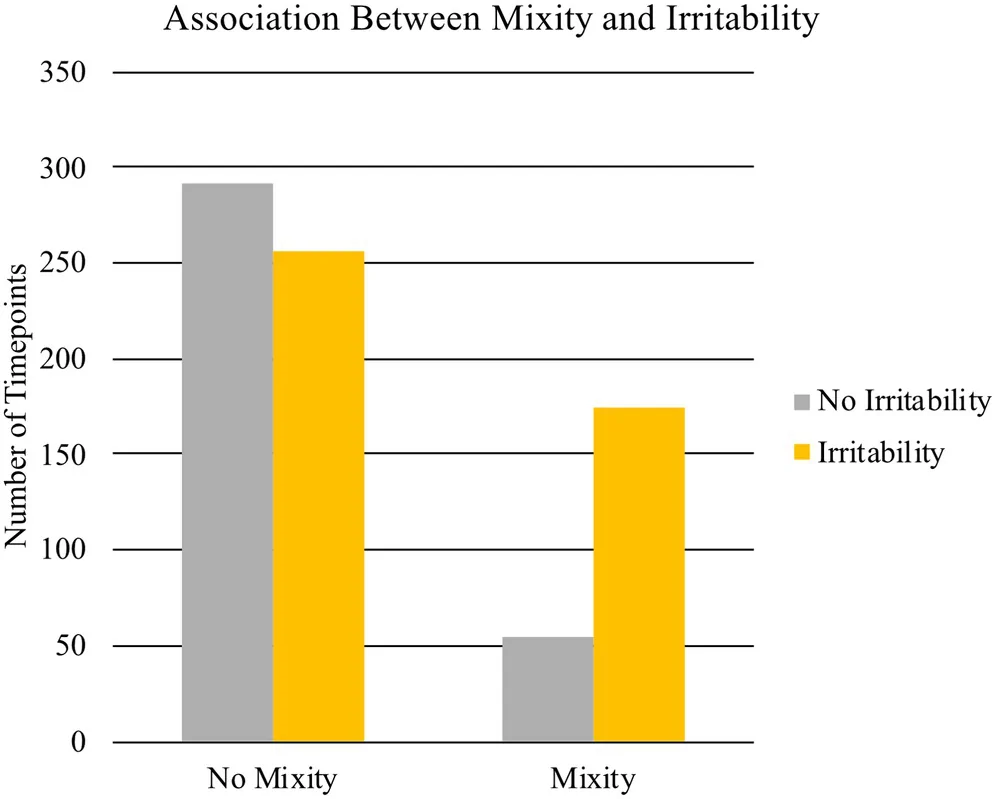
Comparisons of the frequency of irritability where mixity is present vs. absent. This chart shows the results of the crosstabulation of irritability and mixity in sampled timepoints over the study. When mixity is absent, the ratio of no irritability to irritability is almost equal (292:256). However, when mixity is present, the ratio of irritability to no irritability is greater than three (175:54). Thus, within the context of mixity, irritability is significantly more likely and when mixity is absent, irritability is present almost half the time. This shows the association between irritability and mixity, but at the same time these results also demonstrate the general association between irritability and bipolar disorder, in that it is present at least half the time regardless of mixity status.

## 4 Discussion

This exploratory study aimed to examine the relationship between irritability and mixity status in a population of relatively young patients with bipolar disorder. In line with our hypothesis, we found a significant association between mixity and irritability, suggesting a relationship between these two variables.

### 4.1 Prevalence of Mixity

This study analysed data from a group of patients diagnosed with bipolar disorder, and who varied in terms of their phase of illness at the point of entry into the study [30]. It is interesting to note that across the 82 participants examined in this analysis, the vast majority showed the presence of co‐occurring depressive and manic symptoms for at least one timepoint over the 18 months, and over one quarter had mixed symptoms for the majority of the timepoints sampled. It is difficult to compare the findings of this study with others, as the reported prevalence of mixed symptoms vary widely depending on how the population is defined (e.g., bipolar subtypes, including unipolar depression) and how mixed symptoms are defined (e.g., strictly according to DSM mixed features criteria). In addition, our examination was longitudinal and followed patients over a relatively long period of time, whereas many studies take a cross‐sectional approach. However, some comparisons may be drawn from examinations in patients with a broad range of psychopathology and within mood disorders.

A recent retrospective analysis of patient data using a scale similar in application to the LIFE scale used in the present analysis found some degree of mixity (low, mild and high) in 75% of patients admitted to a psychiatric unit who were discharged with a variety of diagnoses, including psychotic, mood, personality and other disorders [36]. In patients with mood disorders, analyses have yielded a wide range in mixity prevalence [18]. For example, in unipolar depression, manic symptoms are prevalent in 38%–47% of patients [37, 38], whereas in bipolar depression the prevalence may be up to 68% [39]. Within the context of mania, co‐occurring depressive symptoms have been shown to occur in 12%–40% of patients [28, 29, 40]. In addition, whilst our cohort sampled relatively young participants with bipolar disorder, global examinations of older adults with bipolar disorder found approximately 70% had mixity [41]. Thus, mixity may be a broad phenomenon, being prevalent not only in the general psychiatric population, but also within mood disorders. The prevalence of mixity in our relatively small cohort therefore seems to be within the ranges of those with mood disorders, despite the fact that the current cohort were undergoing treatment which significantly reduced their symptoms overall. Thus, mixity was confirmed as a prevalent and consistent phenomenon in this cohort.

### 4.2 Irritability and Mixity

Our results indicate a relationship between irritability and mixity status in a dataset which incorporates sampling points over a period of 78 weeks. Thus, this relationship is robust enough to withstand not only an extended period of time but also the impact of a psychological intervention which reduced mood morbidity and irritability over the course of the intervention. Indeed, other examinations in patients with bipolar disorder have shown that dysphoria, which includes irritability, hostility and aggression, occurs in up to 72% of patients with mania [17, 28]. Another longitudinal examination of patients with bipolar disorder found irritability to be present approximately 50% of the time, regardless of affective state [42]. Thus, irritability appears to be a relatively common feature within bipolar presentations, even those that do not meet the criteria for a mixed ‘episode’ per se. In addition, the fact that irritability had a significant relationship with mixity in the current sample is noteworthy, as patients had relatively low levels of mania upon study entry (see Table 2) and this continued to decrease over the course of the study. Thus, irritability may be central and pervasive to mood disorders more generally, and a signal of bipolar disorder, rather than of just mania. As such, irritability could be considered to be a clinical indicator of bipolar illness.

### 4.3 Strengths and Limitations

This study has several notable strengths. Firstly, it examined a group of patients with bipolar disorder longitudinally, with each patient providing approximately ten timepoints of data with scores of subjective irritability. In addition, scores of mood morbidity using the validated LIFE assessment provided a separate subjective examination of the patient's mood morbidity without specific symptom prompts. This separation of mood and irritability measures avoided the conflation that typically occurs when using other clinical assessments such as the Young Mania Rating Scale [43], which includes a clinician rating of irritability. Thus, this dataset provided a unique opportunity to interrogate the relationship between subjective irritability and mixity over time. In addition, this interrogation reflects clinical reality, wherein symptoms of mania and depression overlap, which may not meet the rigid thresholds as described in current diagnostic taxonomies.

Importantly, a key limitation with the current preliminary analysis is that the temporality of the relationship between irritability and mixity or the causal directionality between these two variables cannot be derived. This raises some key questions—is it that increased irritability is a signal for mixity, that is, it heralds the presence of manic symptoms alongside a depressive syndrome? Or does it perhaps signal that when a person experiences mixity, irritability manifests as an epiphenomenon? Future longitudinal studies that sample the temporal relationship of irritability and mood symptoms closely, for example utilising ecological momentary assessments, would provide insights as to whether irritability is driven by underlying mood symptoms or vice‐versa.

In addition to sampling irritability and mood symptoms closely, future examinations of irritability should utilise more validated and comprehensive measures of the construct such as the Brief Irritability Test [2, 44, 45]. This tool assesses the experience of irritability comprehensively and ensures participants and clinicians are not reliant on observable behaviours to indicate irritability (e.g., outbursts or arguments) and that irritability is not conflated with related but distinct phenomena such as anger, agitation and aggression [8]. In addition, as new research adopts this measure for irritability in both clinical and non‐clinical populations, it will allow for more seamless comparisons between populations [2, 45, 46]. This will ultimately assist in understanding irritability's transdiagnostic nature and will further progress our understanding of its relationship to psychopathology more broadly, not just within the context of a single group of disorders such as mood disorders. Further, it will also bridge the gap in understanding between irritability's manifestation in adolescents and adults, thus modelling the phenomenon across the lifespan [13].

### 4.4 Summary and Potential Future Applications

This exploratory analysis of well characterised bipolar patient data has illustrated a relationship between irritability and mixed symptoms and has shown the robustness of this relationship over time and throughout treatment. Thus, the findings confirm the importance of examining irritability in future research, and increasing the focus paid to irritability in clinical practice when monitoring patients with mood disorders. As irritability manifests broadly across the mood spectrum, it may serve as a useful clinical link between poles of mood and between acute episodes of illness. In this way it may be an epiphenomenon of mood disorders, and in particular bipolar disorder. The findings of this study serve as a useful foundation for future, more detailed examinations of the relationship between irritability and other symptoms in mood disorders, including their temporal relationships to one another.

If future longitudinal examinations show that irritability precedes other mood symptoms in mood disorders, then clinicians could intervene earlier and with greater confidence that they are dealing with a possible bipolar illness. This is critical in the context of mood disorders, as they are episodic and recurrent in nature, and manifest in a variety of presentations [47]. Most typically, patients first experience depressive episodes during adolescence and early adulthood and are diagnosed with major depression [48]. When mania first emerges, the person is then assigned a diagnosis of bipolar disorder, with treatment focused on ensuring mood stability and the prevention of future episodes of illness [49]. At the same time, patients are provided with education on how to manage their mood and the early signs to look out for that may indicate a proximal mood episode. If irritability could serve as a clinical indicator that assists clinicians in detecting a patient with incipient bipolar illness, as opposed to unipolar depression, then treatment could proceed down an alternate pathway with the goal of mood stability rather than a focus on specifically lifting mood. Further, if irritability precedes the onset of recurrent episodes, then it could be a useful indicator for patients themselves to recognise when they are at risk of becoming unwell.

## Conflicts of Interest

E.B. has received joint grant funding from the University of Sydney and National Taiwan University (Ignition Grant), The North Foundation and the GYMA Foundation. M.I. reports receiving funding from Gama Foundation. G.S.M. was Editor‐in‐Chief of ANZJP 2010–2021 and is presently College Editor and Editor‐in‐Chief of the British Journal of Psychiatry and advisor to the Editor of Australasian Psychiatry. He has received Wellcome support for GALENOS and grant or research support from the National Health and Medical Research Council, Australian Rotary Health, NSW Health, American Foundation for Suicide Prevention, Ramsay Research and Teaching Fund, Elsevier, AstraZeneca, Janssen‐Cilag, Lundbeck, Otsuka and Servier, and has been a consultant for AstraZeneca, Janssen‐Cilag, Lundbeck, Otsuka and Servier. He is the recipient of an investigator‐initiated grant from Janssen‐Cilag (PoET Study), joint grant funding from the University of Sydney and National Taiwan University (Ignition Grant) and grant funding from The North Foundation. P.B. has received speaker fees from Servier and Janssen. R.B. has nothing to disclose. R.J.P. has received support for travel to educational meetings from Servier and Lundbeck and uses software for research at no cost from Scientific Brain Training Pro.

## Data Availability

Research data are not shared.
